# Physical Exercise As Stabilizer For Alzheimer'S Disease Cognitive Decline: Current Status

**DOI:** 10.2174/1745017901713010181

**Published:** 2017-10-28

**Authors:** Sergio Machado, Alberto Souza de Sá Filho, Matheus Wilbert, Gabriela Barbieri, Victor Almeida, Alexandre Gurgel, Charles V. Rosa, Victor Lins, Alexandre Paixão, Kamila Santana, Gabriel Ramos, Geraldo Maranhão Neto, Flá Paes, Nuno Rocha, Eric Murillo-Rodriguez

**Affiliations:** 1 Laboratory of Physical Activity Neuroscience (LABNAF), Physical Activity Postgraduate Program, Salgado de Oliveira University, , Brazil; Laboratory of Panic & Respiration, Institute of Psychiatry, Federal University of Rio de Janeiro, , Brazil; 2 Physical Activity Science Postgraduate Program, Salgado de Oliveira University, , Brazil; 3 Intercontinental Neuroscience Research Group; 4 Politechnique Institute of Porto, Healthy School, , Portugal; 5 Laboratorio de Neurociencias Moleculares e Integrativas Escuela de Medicina, División Ciencias de la Salud Universidad Anáhuac Mayab,

**Keywords:** Alzheimer’s disease, Unimodal and multimodal training, Cognitive functioning, Physical exercise

## Abstract

**Introduction:**

Mental health decline is one of the main responsible factors for augments in health care costs, and diagnosis of Alzheimer’s disease (AD). Some studies stated physical exercise is useful for reduction in cognitive decline and AD. Moreover, a recent review argued that evidence are scarce due to few studies published and lack of configuration information of exercise protocol, such as intensity and duration of exercise, number of sessions and other relevant data, to allow appropriate assessment.

**Materials and Methods:**

Here, we discussed the possible confounders or factors responsible for these differences and possible neurophysiological mechanisms.

**Results:**

Most studies revealed a possible positive association between physical exercise and cognitive assessments. There are inconsistencies in studies design responsible for varying use of cognitive assessments and different assessments of fitness. However, these studies do not fail to provide evidence about the benefits of exercise, but fail to make it possible because of the lack of dose-response information in AD patients. Physical exercise of moderate intensity should be considered as standard recommendation to reduce cognitive decline, probably due to the improvement in neurodegenerative mechanisms, and the increase in neuroplastic and neuroprotective neurotrophic factors.

**Conclusion:**

Therefore, it is suggested that physical exercise is an important neuroprotective modulator, bringing significant control of the disease and amplifying brain functions.

## 1 INTRODUCTION

Mental health decline is one of the main responsible factors for augments in health care costs, and Alzheimer’s disease (AD) diagnosis [1, 2]. Thus, there is a need of understanding the biological bases and preventive strategies of AD that are crucial for the enhancement of low-cost measures for preventive and treatment programs for AD. For example, despite age is considered a strong risk factor for AD, some epidemiological studies point lifestyle factors out as great responsible factors for prevention and treatment of AD (*e.g.*, physical exercise contributes to significant reduction in age-related risks for cognitive decline and AD) [3, 4].

Some studies stated that physical exercise is useful for reduction in cognitive decline and AD. Moreover, a recent review argued that evidence are scarce due to few studies published and lack of configuration information of exercise protocol, such as intensity and duration of exercise, number of sessions and other relevant data, to allow appropriate assessment [5]. A few studies were conducted examining the effects of physical exercise on cognitive functioning in AD patients, however this issue remains unclear. There is no study examining that question in a critical way. Thus, our objective in this opinion paper is to discuss the potential confounders or mediators that may explain these discrepancies and possible neurophysiological mechanisms. For this purpose, we did an electronic search on Pubmed and ISI Web of Knowledge data bases for randomized controlled trails and systematic and metaanalytical review studies on that theme.

Within this context, a few studies were found to examine the effects of physical exercise on cognitive functions of AD patients. Yaguéz *et al.* [6] proposed a new exercise program for medicated AD patients, the “Brain Gym”, composed of stretching, mobility and isometric exercises. After 6 weeks of 2 hours of non-aerobic exercise, the exercise group showed significant improvement in sustained attention, visual and working memory compared to the control group, which received coping strategies and psychological care. Vreugdenhil *et al.* [7] demonstrated that a progressive multimodal training (strength and balance exercises and 30-minute walking at 65-75% HRmax) combined with medication, performed for 16 weeks, was more effective on augmentation of multiple cognitive performance than medication isolated in mild to moderate AD.

In line with this, Andrade *et al.* [8] corroborated Vreugdenhil *et al.* [7] findings, demonstrating that 60 minutes of progressive dual-task, 3 times per week, *i.e.*, cognitive stimuli (*i.e.*, speak animals, fruits, flowers, or people names, count backward, or nominate figures and colors) and motor stimuli (*i.e.*, walking, bouncing a ball up and down a stairway, strength exercises) for 16 weeks was effective on cognitive functioning in mild to moderate medicated AD patients compared to controls (*i.e.*, without any activity). Such effects suggest that physical exercise is an important neuroprotective modulator, *i.e.*, physical conditioning may bring significant control of the disease, amplifying brain functions. Despite the correct methodological design, and the prominent effects of the exercise, the evidence fails in the description of the exercises chosen for the exercise program, as well as the intensity and duration of the exercises.

In general, these studies do not fail to provide evidence about the benefits of exercise; they fail to make it possible to understand the dose-response in patients with AD. Winchester *et al.* [9], examined the association between exercise and cognitive functioning in 104 mild AD patients, over 1 year. In this study, active and sedentary patients were divided based on their profiles. Patients that spent time engaged in physical activity showed a reduction in global cognitive decline over time (Mini-Mental State Examination – MMSE total score), while sedentary patients had a significant decline in global cognitive functioning. In the group of active patients, those who engaged in walking for more than 2 hours per week demonstrated a significant improvement in global cognitive functioning. Venturelli *et al.* [10] verified the effects of 24 weeks of walking, during 4 days per week, additional to medication on cognitive decline of institutionalized severe AD patients. Exercise group did not show any significant difference in global cognitive functioning, however the same was not found for control group (*i.e.*, medicated), which demonstrated a reduction by 47% in global cognitive functioning. Findings indicate that aerobic exercise can stabilize the progressive cognitive decline in nursing home AD residents. More recently, Arcoverde *et al.* [8] investigated the effects of 16 weeks aerobic exercise (*i.e.*, walking) added to medication on mild AD (20 elderly with dementia). Training was performed twice a week for 3 months, with 20 minutes at 60% VO2max. Significant improvement in global cognitive functioning was observed in exercise group when compared to control group, which maintained only pharmacological treatment and showed a decline in global cognitive functioning, specifically in executive functions. In addition, the effect size was measured and presented values classified as large (1.17 to 1.58) according to Cohen index for cognitive responses (exercise group). The limitation of this study was the inclusion of two patients with mixed dementia, which for us can be seen as a partial confounding factor, not disqualifying the study as positive evidence of exercise.

Some neurobiological mechanisms could explain the findings of these studies. Most of them revealed a possible positive association between physical exercise and cognitive assessments. Inconsistency in studies design is responsible for varying use of cognitive assessments; different assessments of fitness (objective *vs* self-reported hours of exercise practice); and diverse classification for high/low exercise levels across studies. Other studies point out to these limitations stating their potential confounds related to improvement in both exercise and cognitive functioning, such as reduction in depressive symptoms, as well as increase in social interaction and cognitive engagement [11], and an increase in self-efficacy that has generally not been analyzed or satisfactorily controlled for.

Lack of causality of relationship and exercise cannot be interrupted due to other confounding morbidity that can also influence on cognitive functioning (*e.g.*, vascular disease). Randomized controlled studies are the gold standard to establish causality, however some biases can interfere on several methodological factor, such as the choice of measurements and population, baseline variation, and control conditions configuration (*e.g.* no social or cognitive stimulation), and possible non-appraised confounders or mediators, *e.g.* mood.

Some neurophysiological hypothesis could be discussed. Brain activity is positively associated with an increase in oxygen and glucose consumption and an augment in regional cerebral blood flow (rCBF). Exercise is associated with augment of rCBF in various brain areas [12], where adenine nucleotides participate in rCBF control sites. Within this context, a study examined if circulating nucleotides and derivatives, from skeletal active muscles, reach deleterious levels for brain metabolism in the arterial blood, through body metabolic communication interceded by circulating purine compounds. Levels of adenosine triphosphate (ATP), a powerful vasodilator, also increase during exercise and this may also be a mechanism implicated in the regulation of rCBF [13]. Brain perfusion is also dependent on nitric oxide (NO), and exercise assists in endothelial synthesis of NO, thus improving angiogenesis and regulation of rCBF [14, 15]. Increased neurotrophic factor of neural growth, with favorable repercussion for cerebral plasticity, has also been described, where exercise acts as protective agent and can be clarified through hormesis theory. This theory states that low doses of toxins and/or radiation could influence beneficially on the body [16]. The study of Radak and colleagues [12, 16] argued that reactive oxygen species (ROS) would be part of hormesis theory, with positive effects of regular exercise based on their capability to produce ROS. The production of ROS after exercise practice acts as an inductor of anti-oxidants, thereby reducing the incidence of oxidative stress related diseases [12]. The findings demonstrated that physical exercise is related to attenuation of cognitive decline in AD. A temporary improvement of various cognitive functions (*e.g.*, attention and executive functions) and attenuation of a neurodegenerative progression are observed in the studies, probably due to the improvement in neurodegenerative mechanisms, and the increase in neuroplastic and neuroprotective neurotrophic factors.

Therefore, physical exercise of moderate intensity should be thought as standard recommendation to reduce cognitive decline. Given the relevance of the theme and the challenges to AD treatment, physical exercise represents a beneficial non-pharmacological intervention for these patients. There is a need of new controlled studies to ascertain which intervention strategies would be more appropriate as a form of adjuvant treatment in AD.
